# Why Do We “Like” on WeChat Moments: The Effects of Personality Traits and Content Characteristics

**DOI:** 10.3389/fpsyg.2022.772547

**Published:** 2022-02-24

**Authors:** Chun Zheng, Xingyu Song, Jieyun Li, Yijiang Chen, Tingyue Dong, Sha Yang

**Affiliations:** ^1^Department of Psychology, School of Education and Psychology, Southwest Minzu University, Chengdu, China; ^2^School of Psychology, Central China Normal University, Wuhan, China

**Keywords:** social media, WeChat Moments, giving “Like” feedback, emotional valence, personal relevance, personality traits

## Abstract

To probe the motivational roles of hedonic gratification and social gratification in giving “Like” feedback on social media, we developed a set of novel pictures to simulate WeChat Moments. We subsequently examined how the personality trait of extraversion and stimulus content characteristics (e.g., emotional valence, personal relevance) influenced “Liking” behavior. A 2 (extraversion: extrovert group vs. introvert group) × 3 (emotional valence: positive vs. neutral vs. negative) × 2 (personal relevance: personally relevant vs. personally irrelevant)-mixed experimental design was applied to data obtained from 56 WeChat Moments users. These participants included 28 individuals with the highest extraversion scale scores (the extrovert group), and 28 individuals with the lowest extraversion scale scores (the introvert group), according to the NEO Five-Factor Inventory. Briefly, participants observed pictures on an interface similar to that of WeChat Moments and were given the option to “Like” each picture. “Like” rates and response time were then compared across groups and conditions by applying a mixed-design analysis of variance. Pearson's correlation coefficients were calculated to explore relationships between the “Like” rates under each condition and the scores for each personality trait. Compared with the neutral pictures, the positive and negative pictures were “Liked” more and less frequently, respectively (*F*_2, 108_ = 46.22, *p* < 0.001). Compared with the poster-unrelated pictures, the personally related pictures were “Liked” more frequently (*F*_1, 54_ = 19.54, *p* < 0.001). In the extrovert group, the frequency of “Likes” given to unrelated negative content positively associated with neuroticism (*r* = 0.42, *p* = 0.025) and negatively associated with conscientiousness (*r* = −0.46, *p* = 0.014). No correlations were observed in the introvert group. Compared with not giving “Like” feedback, participants gave “Likes” to positive and negative pictures more quickly (*p* = 0.035) and slowly (*p* < 0.001), respectively.These results support the hypothesis that hedonic gratification and social gratification motivate “Like” feedback for positive content and personally related content, respectively. “Liking” behavior was not affected by extraversion, but was related to neuroticism and conscientiousness. Content-related differences in time intervals for giving “Like” feedback in this study suggest that people do not hesitate to give “Like” feedback to positive content on WeChat Moments, yet linger in deciding to give “Like” feedback to negative content.

## Introduction

With the booming development of social media platforms such as WeChat Moments, QQ Zone, Microblog, Facebook, Twitter, and Instagram, the “Like” button has become one of the easiest and most rapid response options to enable users to communicate through a single click. Consequently, the “Like” option has become immensely popular (Sumner et al., 2018). “Liking” behavior on social media platforms is also an important indicator of how users give feedback, obtain feedback, and make social comparisons. For example, on WeChat Moments (a popular social media platform in China, hereafter referred to as “Moments”), users can “Like” content posted by their friends without any implicit, ambiguous, or double-entendre implications. Users can also review “Like” feedback from their friends after posting content, and “Like” feedback from mutual friends after their friends post content. The number of “Likes” received from friends are readily observable below each post. Thus, “Liking” is standardized, visual, and quantifiable feedback, respectively. Compared with face-to-face or peer-to-peer social contact, “Liking” represents a novel type of communication.

To investigate why people provide “Like” feedback on social media, a number of studies have been conducted. Motivations for “Liking” behavior on social media have been discussed within the theoretical framework of uses and gratifications (U&G) (Kim, 2014; Hayes et al., 2016; Lee et al., 2016; Gan and Chunmei, 2017; Ozanne et al., 2017; Hossain et al., 2019; Shao and Kwon, 2019; Wang et al., 2019), which is a well-known and effective approach for understanding why and how people use media (Ruggiero, 2000). Previous studies have empirically validated the hypothesis that behavioral intentions of “Liking” are predicted by three aspects of gratification, hedonic, social, and utilitarian (Gan and Chunmei, 2017; Ozanne et al., 2017; Hossain et al., 2019). Hedonic gratification refers to enjoyment (Lee et al., 2016; Gan and Chunmei, 2017; Hossain et al., 2019; Shao and Kwon, 2019), entertainment (Kim, 2014; Ozanne et al., 2017), and passing time (Lee et al., 2016). Social gratification refers to social support (Hayes et al., 2016; Gan and Chunmei, 2017), interpersonal relationships (Lee et al., 2016), social interactions (Hossain et al., 2019), bonding (Ozanne et al., 2017), and socialization (Kim, 2014). Meanwhile, utilitarian gratification refers to seeking information (Kim, 2014; Gan and Chunmei, 2017; Ozanne et al., 2017; Hossain et al., 2019) and expression (Kim, 2014; Shao and Kwon, 2019). These factors have been proved as motivations which significantly predict “Liking” behavior either directly or indirectly based on intention, and this in turn decides “Liking” behavior (Shao and Kwon, 2019).

In addition to the U&G approach, scholars have considered other theories to account for factors that empirically influence “Liking” behavior on social media. Based on the theory of planned behavior (TPB), some studies have shown that relational facilitation, self-presentation, and metacommunication are key drivers of “Liking” activity on Facebook (Sumner et al., 2018; Dhir et al., 2019). By integrating guanxi theory and the affective response model, another study has indicated that guanxi cues (i.e., mianzi giving, renqing, and ganqing) positively and indirectly affect the intention of Chinese people to “Like” on the Moments platform (Zhao and Zhang, 2020). Since relational facilitation, self-presentation, metacommunication, and guanxi have also been examined in interpersonal relationships (Sumner et al., 2018; Dhir et al., 2019; Zhao and Zhang, 2020), it is consistent for these factors to be considered for their social value in influencing “Liking” behavior in social media.

It has been demonstrated that hedonic gratification, social gratification, and utilitarian gratification can motivate “Like” intentions (Chin et al., 2015; Sun et al., 2016; Dhir et al., 2019; Hossain et al., 2019; Shao and Kwon, 2019; Wang et al., 2019). According to TPB, individual behavior is influenced by behavioral intention (Ajzen, 1991). Consequently, the stronger the “Like” intention of users, the greater the chance that “Liking” behavior will be exhibited. However, it is important to note that “Like” intention does not universally lead to actual “Liking” behavior (Sun et al., 2016). In addition, given that an unobtrusive measure of actual “Liking” behavior is not obtainable, self-reporting data (Lee et al., 2016; Gan and Chunmei, 2017; Hossain et al., 2019; Shao and Kwon, 2019; Li and Wang, 2021) or information from interviews (Hayes et al., 2016; Ozanne et al., 2017) have been used to obtain individuals' recollections of past behaviors. Yet, while “Like” intentions have been used to predict past “Liking” behavior, it remains for actual “Liking” behavior to be investigated to reveal how gratifications drive actual “Liking” behavior on social media. Chin et al. (2015) conducted a field experiment to investigate the relationship between motivations and actual “Liking” behavior. They developed 32 different combinations of content posted on Facebook while controlling five variables: hedonic, utilitarian, affiliation, compliance, and conformity motivations. Each content posted on Facebook was displayed to participants, and the participants subsequently responded on survey forms. The resulted showed that the five motivations examined all had a positive impact on the participants' attitudes toward “Liking,” which in turn had a positive impact on their “Like” intention. Furthermore, it was observed that “Like” intention had a positive impact on actual “Liking” behaviors (Chin et al., 2015).

The design of the field experiment described above provides a useful reference for investigations of the relationships between motivations and actual “Liking” behavior. However, there are two aspects that need to be considered in future studies. One is that Chin et al. (2015) used structural equation modeling to examine the relationships among five motivations, attitudes toward “Liking,” “Like” intentions, and “Liking” behavior. However, it remains unknown whether these motivations represent independent variables that drive users to give or not give “Like” feedback on social media. The second aspect to consider is that utilitarian gratification was confused with hedonic gratification in the study by Chin et al. (2015), since four types of positive and negative narratives were designated as hedonic and utilitarian. Utilitarian gratification includes expression and information seeking as motivations of “Liking” behavior, and these can vary greatly among individuals. Consequently, it is not feasible to control utilitarian gratification as a variable of content posted on social media. Therefore, in the design of the present study, only hedonic gratification and social gratification were examined, with different types of simulated Moments content developed to address these two motivation variables.

It has been suggested that the motivations for using social media can vary according to individuals' personality traits (Papacharissi and Mendelson, 2011). However, to date, few studies have investigated the relationship between personality traits and “Liking” behavior on social media. Moreover, among the studies which have performed these investigations, their results are not consistent. For example, Kabadayi and Price (2014) observed that extraversion, neuroticism, and openness to experiences indirectly influenced “Liking” behavior. In another study, affective attitudes toward Facebook “Liking” were positively predicted by extraversion and agreeableness, while openness to experiences was a negative predictor (Lee et al., 2016). In a more recent study, agreeableness and conscientiousness, yet not extraversion, had positive and negative effects on “Liking” behavior, respectively (Li and Wang, 2021). In terms of social media use, extraversion has been associated with a greater number of Facebook friends (Tazghini and Siedlecki, 2013). Moreover, some studies have found that extraversion is the most significant personality trait predictor for use of social media (Wilson et al., 2010; Gosling et al., 2011). Extraversion describes a person's tendency to be sociable and his/her ability to experience positive emotions (Butt and Phillips, 2008). Thus, extraversion is related to both social gratification and hedonic gratification. In the present study, extraversion will be evaluated as a personality trait variable to examine whether extroverts or introverts exhibit greater “Liking” behavior on social media.

Previous studies have used questionnaires, interviews, and field experiments to investigate hedonic and social gratification as motivations of “Liking” behavior on social media. However, it remains unclear whether “Liking” behavior is related to extraversion as a personality trait. Moreover, to date, no experimental studies have strictly manipulated independent variables and controlled irrelevant variables to confirm the effects of extraversion, hedonic gratification, and social gratification on actual “Liking” behavior. Therefore, the aim of this study was to compare the responses of two groups of participants who received high (extrovert group) vs. low (introvert group) scores for the extraversion trait. Both groups were shown images of simulated Moments content that exhibited different emotional valences (positive vs. neutral vs. negative) and different personal relevance to the poster (personally relevant vs. personally irrelevant). The participants were given the option to “Like” or “No-like” the pictures. Similar to the field experiment study conducted by Chin et al. (2015), emotional valences were designed to represent hedonic gratification in the present study. Regarding social gratification, Chin et al. (2015), affiliation motivation was designated according to whether or not the posted content was related to the originators' daily lives or mood changes. Furthermore, estimates of the affiliation motivation of the participants were based on scoring of items such as, “the content of this post allows me to be informed about poster's living/mood/recent status” (Chin et al., 2015). Therefore, personal relevance to the poster was designed to represent social gratification in the present study. According to U&G theory and the results of previous studies, the following hypotheses were established for the present study. Hypothesis 1: Simulated positive images from Moments will receive more “Likes” than neutral and negative images. Hypothesis 2: Pictures accompanied by personally relevant captions will receive more “Likes” than pictures accompanied by personally irrelevant captions. Hypothesis 3: Participants in the extrovert group will “Like” simulated Moments images more often than participants in the introvert group.

## Methods

### Participants

A total of 155 college students completed the NEO Five-Factor Inventory (Luo and Dai, 2011). Among these students, 28 were included in an extrovert group (3 males, 25 females), and 28 were included in an introvert group (4 males, 24 females). The two groups were age-matched (extrovert group: 19.00 ± 1.05 years old, introvert group: 19.39 ± 0.99 years old, *t*_54_ = −1.44, *p* = 0.157, Cohen's *d* = 0.38) and all of the participants exhibited normal or corrected visual acuity. Informed consent forms were collected from all of the participating students prior to the start of the experiment, and each received remuneration at the end of the experiment.

### Stimuli and Tool

#### Images Simulating WeChat Moments

**Figure 1:**
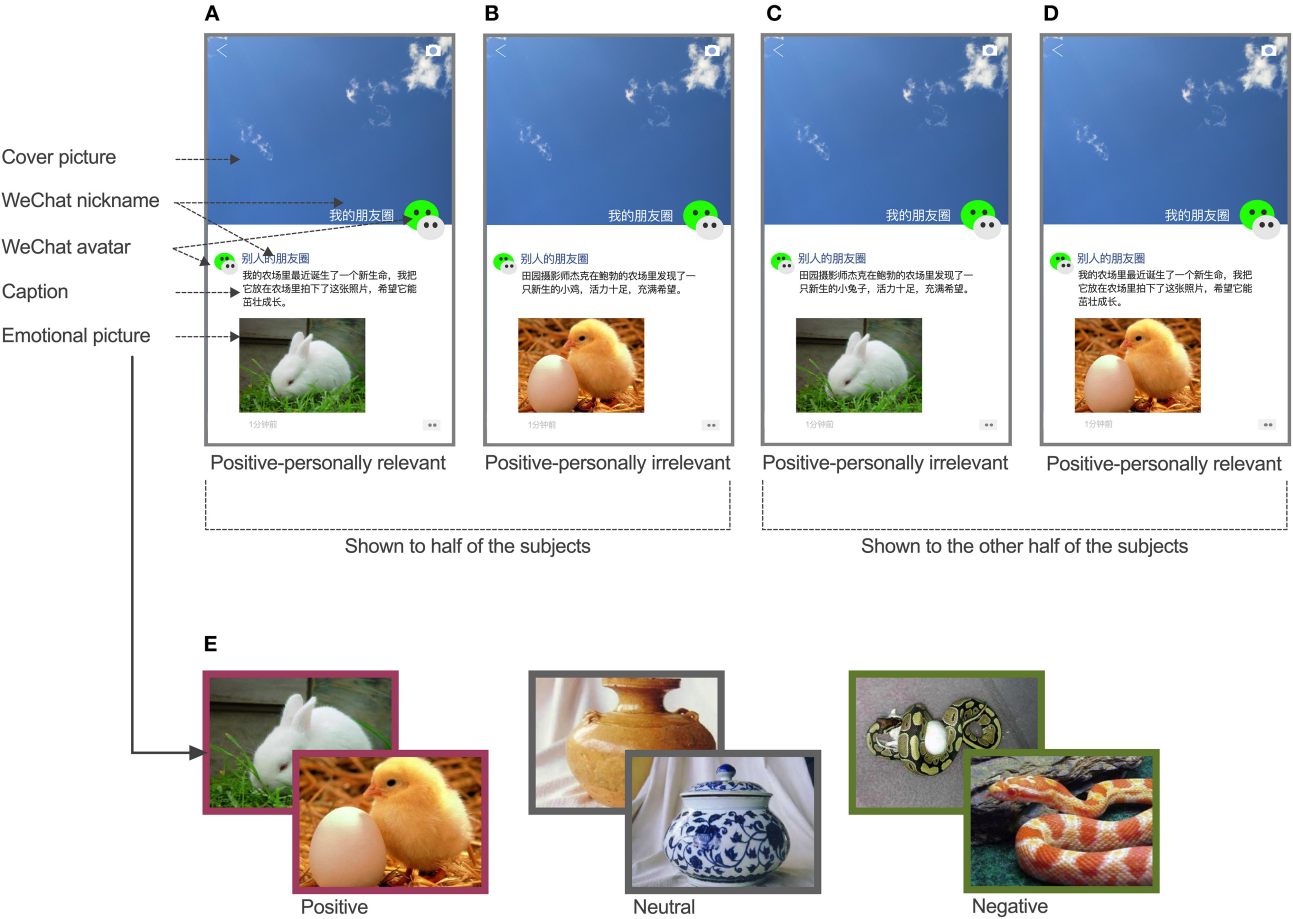
Examples of simulated Moments images and emotional pictures. A pair of positive emotional pictures of similar situations were matched with two types of captions, personally relevant captions **(A,D)** or personally irrelevant captions **(B,C)**. Half of the participants in the extrovert and introvert groups were shown **(A)** and **(B)** during the experimental task, while the other half of the participants were shown **(C)** and **(D)**. **(E)** Examples of emotional pictures. Three sets of positive, neutral, and negative pictures, respectively. “我的朋友圈”: “My Moments”; “别人的朋友圈”: “Other's Moments”; **(A)**: “我的农场里最近诞生了一个新生命, 我把它放在农场里拍下这张照片, 希望它能茁壮成长。” (“A new life was born recently. I put it on my farm and took this picture. I hope it will thrive.”); **(B)**: “田园摄影师杰克在鲍勃的农场里发现了一只新生的小鸡, 活力十足, 充满希望。” (“Jack, a field photographer, found a newborn chick on Bob's farm with great vigor and hope.”); **(C)**: “田园摄影师杰克在鲍勃的农场里发现了一只新生的小兔子, 活力十足, 充满希望。” (“Jack, a field photographer, found a newborn rabbit on Bob's farm with great vigor and hope.”); **(D)**: “我的农场里最近诞生了一个新生命, 我把它放在农场里拍下了这张照片, 希望它能茁壮成长。” (“A new life was born recently. I put it on my farm and took this picture. I hope it will thrive.”). (Images retrieved with permission from: Chinese Affective Picture System).

To simulate real WeChat Moments, the picture materials used in this study included screenshots of images from actual online Moments accounts. The images were subsequently processed so that each image consisted of a consistent cover picture, two WeChat avatars (viewer and poster), two nicknames (viewer and poster), an emotional picture, and a caption (Figures 1A–D). The emotional valence of each emotional picture (positive vs. neutral vs. negative) and the personal relevance of each poster's captions (personally relevant vs. personally irrelevant) were independent variables that were manipulated. To reduce interference from irrelevant stimuli, the cover of each simulated Moments image was set as a blue sky, while the avatars of the poster and viewer were represented as WeChat logos. The nicknames of the viewers were “My Moments” and the posters' were labeled, “Other's Moments.” A total of 120 simulated Moments images were divided into two sets of 60 images each. The emotional pictures were repeated in both sets, but if one picture was paired with personally relevant captions in one set, it was paired with irrelevant captions in the other set. In each set, there were six conditions (positive-personally relevant, neutral-personally relevant, negative-personally relevant, positive-personally irrelevant, neutral-personally irrelevant, and negative-personally irrelevant), and each condition had 10 pictures. Half of the participants were shown one set of simulated Moments images, while the other half of the participants were shown the other set of simulated Moments images. Consequently, each picture was presented only once in the experiment.

#### Emotional Pictures

A total of 20 positive, 20 neutral, and 20 negative emotional pictures were selected from the Chinese Affective Picture System (CAPS) database according to the emotional valence scores assigned to each (Bai et al., 2005). Each type of emotion contained 10 pairs of pictures representing similar situations, resulting in a total of 60 selected pictures. Representative pictures are shown in Figure 1E. Additional college students (10 males, 10 females; mean age: 20.60 ± 0.94 y) were recruited to score the emotional valence of the selected emotional pictures using a 9-point scale (1 = very negative, 5 = neutral, 9 = very positive). Significant differences were observed among the emotional valence scores received for the positive (7.14 ± 0.53), neutral (5.79 ± 0.47) and negative (2.81 ± 0.53) pictures (*F*_2, 57_ = 379.26, *p* < 0.001, partial η^2^ = 0.93).

#### Captions

Four college students wrote a personally relevant and a personally irrelevant caption for each emotional picture based on its content. The former were related to the poster's personal life and included the terms “I” or “my.” In contrast, the latter were not related to the poster's personal life and included terms such as “others,” “everyone,” or others' names. For each pair of emotional pictures for similar situations, one was paired with a personally irrelevant caption, while the other was paired with a personally relevant caption. Both captions described the same topic. Figures 1A–D, show representative simulated Moments images of emotional pictures of a similar situation with personally relevant and personally irrelevant captions. Half of the participants in each of the extrovert and introvert groups were shown in Figures 1A,B in the formal experiment, while the other half of the two groups were shown in Figures 1C,D for the task.

#### NEO Five-Factor Inventory

The 60-item NEO Five-Factor Inventory (NEO-FFI) was previously compiled by Costa and McCrae (Costa and McCrae, 1992; Mccrae and Costa, 2004) to measure five basic personality factors: neuroticism, extraversion, openness, agreeableness, and conscientiousness. Each scale includes 12 items for a total of 60 items. A Chinese translation of the NEO-FFI has been widely used (Luo and Dai, 2011). Cronbach's α coefficients for each scale among the Chinese college students were 0.77 for neuroticism, 0.78 for extraversion, 0.63 for openness, 0.72 for agreeableness, and 0.74 for conscientiousness (Yao and Liang, 2010).

### Experimental Procedure

A mixed-design of 2 (extraversion: extrovert group vs. introvert group) × 3 (emotional valence of simulated Moments images: positive vs. neutral vs. negative) × 2 (poster's personal relevance: personally relevant vs. personally irrelevant) was used. Extraversion was the between-subjects variable, while emotional valence and personal relevance were within-subjects variables. “Like” rates and “Like”/“No-like” response times were dependent variables.

#### Questionnaire Testing and Screening

The NEO-FFI was completed by groups of participants before the formal experiment. A total of 155 valid questionnaires were collected. Scores for each of the five scales were calculated separately for each participant. For the formal experiment, the participants with the highest extraversion scale scores (*n* = 30) were included in an extrovert group, while participants with the lowest extraversion scale scores (*n* = 30) were included in an introvert group. Behavioral data for two participants in the extrovert group and two participants in the introvert group did not match with their questionnaire scores, so data from these four individuals were excluded from analysis. Consequently, a total of 28 participants in the extrovert group and 28 participants in the introvert group were included in the final analysis. The differences in extraversion scores between the two groups were very significant (extrovert group: 45.07 ± 2.60, introvert group: 31.07 ± 4.60, *t*_54_ = 14.02, *p* < 0.001, Cohen's *d* = 3.75).

#### Formal Experiment

The experiments were conducted in a quiet room and each participant performed their tasks individually. The experimental procedure was presented by E-prime 2.0 (Psychology Software Tools, Inc., Pittsburgh, PA, USA). Briefly, each participant was asked to respond with or without a “Like” keystroke to simulated Moments images presented on a screen. There was no requirement for the participants to respond as quickly as possible. After understanding the instructions, the participants pressed the space bar to start the experiment. Each trial started with a white “+” gaze point presented on a gray screen for 350 ms. This was followed by presentation of a simulated Moments image in the center of the gray screen. If the participant wanted to “Like” the image, they were instructed to press “F.” If the participant did not want to “Like” the image, they were instructed to press “J.” A blank gray screen was subsequently displayed for 1,500 ms before the next trial was started. A total of 60 trials were completed by each participant. At the end of the experiment, the purpose of the experiment was explained to each participant and they were asked to keep the experiment confidential.

### Data Analysis

All statistical analyses were performed by using SPSS 19.0 (SPSS, Inc., Chicago, IL, USA). The independent samples *t*-test was used to compare age and extraversion scores between the extrovert and introvert groups. To compare the emotional valences of the emotional pictures, one-way analysis of variance (ANOVA) between subjects was performed.

The experiments conducted recorded whether the participants “Liked” the simulated Moments images presented, and when they “Liked” or “No-liked” the pictures. These data were subsequently analyzed. “Like” rate was equal to the number of trials “Liked” for a given condition divided by the total number of trials for that same condition. “Like”/“No-like” response time was also recorded and was defined as the time between presentation of a simulated Moments image and the reaction recorded by a participant. If a participant's “Like”/“No-like” rate was zero, then the participant's “Like”/“No-like” reaction time was calculated as the average of all the participants' “Like”/“No-like” response times under this condition.

“Like” rate data were entered into a 2 (extrovert vs. introvert groups) × 3 (positive vs. neutral vs. negative emotional valence) × 2 (personally relevant vs. personally irrelevant) mixed-design ANOVA, with repeated measures on the last two factors. Similarly, “Like”/“No-like” response times were entered into a 2 (extrovert vs. introvert groups) × 2 (“Like” vs. “No-like”) × 3 (positive vs. neutral vs. negative emotional valence) × 2 (personally relevant vs. personally irrelevant) mixed-design ANOVA, with repeated measures on the last three factors. If the main effect (greater than or equal to three levels) or interaction effect of ANOVA was significant, a *post hoc* test or simple effect analysis was performed.

Pearson's correlations were computed to explore relationships between the “Like” rates and the scores for each dimension of the NEO-FFI. A Greenhouse-Geisser correction was applied to the ANOVA results when the data did not meet the spherical assumption, with the *df* value still reporting the result before correction. The effect size of ANOVA was reported by using partial eta squared (partial η^2^). Cohen's *d* was used as a *t*-test effect measure. Bonferroni correction was used for both *post hoc* test and simple effects analyses. The significance level of the results was set at *p* < 0.05.

## Results

### “Like” Rates

**Figure 2:**
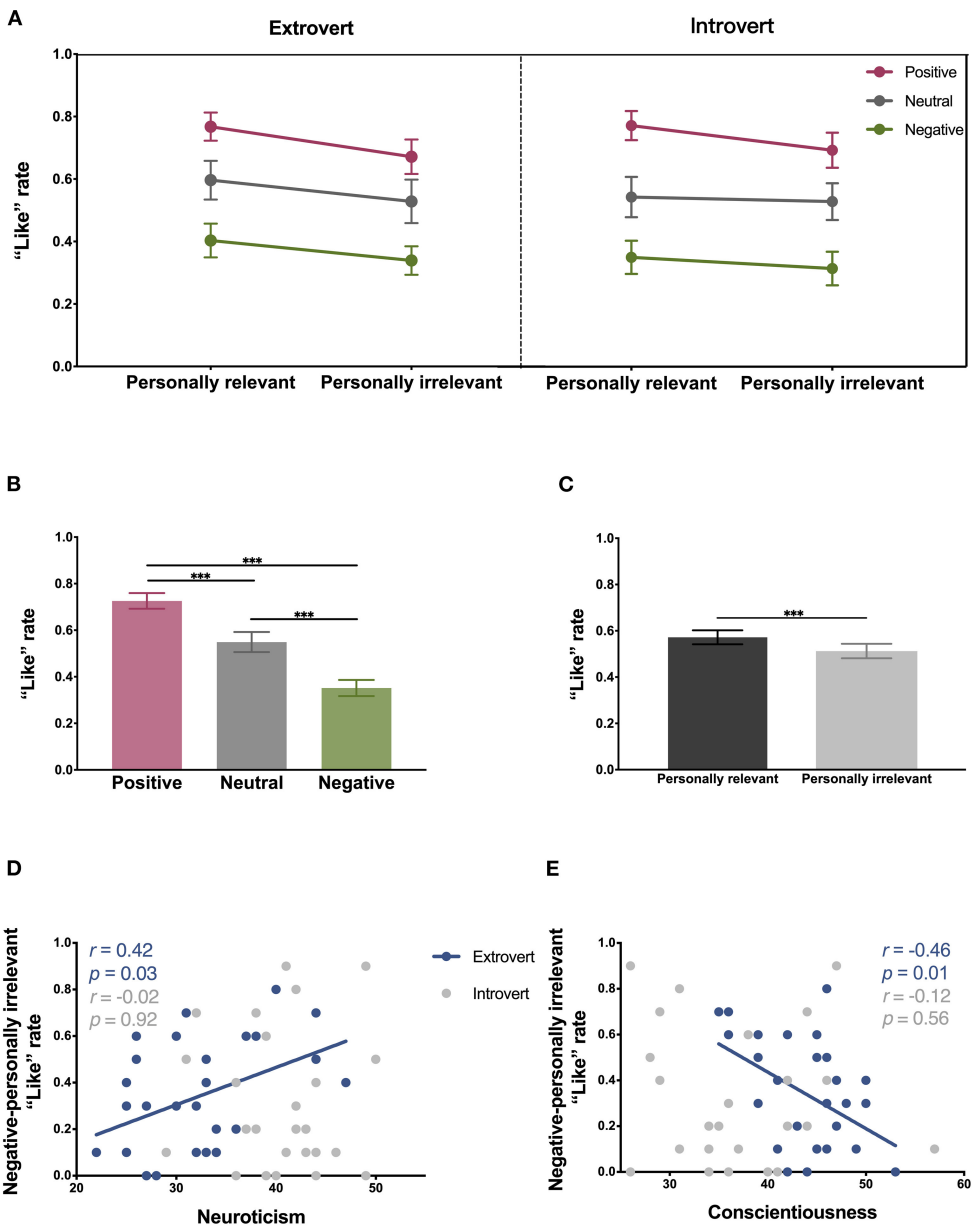
“Like” rates and their correlation with personality traits. **(A)** “Like” rates for the extrovert and introvert groups for the six conditions examined: positive-personally relevant, neutral-personally relevant, negative-personally relevant, positive-personally irrelevant, neutral-personally irrelevant, and negative-personally irrelevant. Three-factor ANOVA results demonstrate that the main effect of emotional valence ($F_{2,108}=46.22,\;p<0.001,\text{ partial }\eta^{2}=0.46$) and the main effect of personal relevance ($F_{1,54}=19.54,\;p<0.001,\;{\text{partial η}}^{2}=0.27$) were significant. **(B)** The main effect of emotional valence on “Like” rate (positive vs. neutral: Cohen's *d* = 0.63, positive vs. negative: Cohen's *d* = 1.49, neutral vs. negative: Cohen's *d* = 0.69). **(C)** The main effect of personal relevance on “Like” rate (relevance vs. irrelevance: Cohen's *d* = 0.26). **(D)** Correlations between “Like” rates and neuroticism scores for the negative-personally irrelevant condition. **(E)** Correlations between “Like” rates and conscientiousness scores for the negative-personally irrelevant condition. Error bars indicate standard errors. ***p < 0.001.

“Like” rates were determined for the six types of simulated Moments images presented to the extrovert and introvert groups in this study (Figure 2A). Three-factor ANOVA results demonstrate that the main effect of emotional valence was significant ($F_{2,108}=46.22,\;p<0.001,\text{ partial }\eta^{2}=0.46$) (Figure 2B), indicating that the simulated Moments images with different emotional valences received different “Like” rates. The main effect of personal relevance was also significant ($F_{1,54}=19.54,\;p<0.001,\;{\text{partial η}}^{2}=0.27$) (Figure 2C), with the simulated Moments images with personally relevant captions receiving a higher “Like” rate than the pictures with captions without personal relevance. In contrast, neither the main effect of extraversion, nor the interaction effect, were significant (main effect of extraversion: $F_{1,54}=0.09,\;p=0.769,\;{\text{partial η}}^{2}=0.00$; interaction effect of extraversion × emotional valence: $F_{2,108}=0.24,\;p=0.786,\;{\text{partial η}}^{2}=0.00$; interaction effect of extraversion × personal relevance: $F_{1,54}=1.53,\;p=0.221,\;{\text{partial η}}^{2}=0.03$; interaction effect of emotional valence × personal relevance: $F_{2,108}=1.11,\;p=0.332,\text{ partial }\eta^{2}=0.02$; interaction effect of extraversion × emotional valence × personal relevance: $F_{2,108}=0.15,\;p=0.857,\;{\text{partial η}}^{2}=0.00$). Thus, no difference in “Like” rate was observed between the extrovert and introvert groups.

A *post hoc* analysis of the main effect of emotional valence identified a significant difference in the “Like” rates of positive (0.73±0.25), neutral (0.55±0.32), and negative (0.35±0.26) simulated Moments images (*ps* < 0.001, positive vs. neutral: Cohen's *d* = 0.63, positive vs. negative: Cohen's *d* = 1.49, neutral vs. negative: Cohen's *d* = 0.69) (Figure 2B). A pairwise comparison of the main effect of personal relevance also showed that the “Like” rates of the simulated Moments images of personal relevance (0.57±0.23) were higher than those for the simulated Moments images of personal irrelevance (0.51±0.23) (*p* < 0.001, Cohen's *d* = 0.26) (Figure 2C).

### Correlation Between “Like” Rates and Personality Traits

Correlation coefficients between the “Like” rates of the six types of simulated Moments images and the scores from each dimension of the NEO-FFI were calculated. No significant correlations were observed when all of the participants were included in the correlation analysis (*ps* > 0.05). However, when the correlations were calculated separately for the extrovert and introvert groups, the “Like” rates in the extrovert group for the negative-personally irrelevant condition were found to positively correlate with neuroticism scores (*r* = 0.42, *p* = 0.025) (Figure 2D) and negatively correlate with conscientiousness scores (*r* = −0.46, *p* = 0.014) (Figure 2E). In contrast, no significant correlations were identified in the introvert group.

### “Like”/“No-Like” Response Time

Participants in the extrovert and introvert groups responded to the six types of simulated Moments images with “Like” or “No-like” feedback. Four-factor ANOVA results showed that the main effect of emotional valence was significant ($F_{2,108}=5.28,\;p=0.007,\;{\text{partial η}}^{2}=0.09$). Thus, differences were observed in the duration of the participants' decisions to “Like” or “No-like” the simulated Moments images with different emotional valences. In particular, whether to “Like” × emotional valence interaction effect was significant ($F_{2,108}=6.27,\;p=0.003,\;{\text{partial η}}^{2}=0.10$). These results indicate that the duration of a participant's decision to “Like” the simulated Moments images that have different emotional valences differed from the duration of the decision to “No-like” those images. The emotional valence × personal relevance interaction effect was also found to be significant ($F_{2,108}=3.41,\;p=0.044,\;{\text{partial η}}^{2}=0.06$). The latter result indicates that the duration of the decision to give or not give “Like” feedback to the pictures with different emotional valences differed for the personally relevant vs. personally irrelevant conditions. No other main effect or interaction effects were observed (*ps* > 0.05).

**Figure 3:**
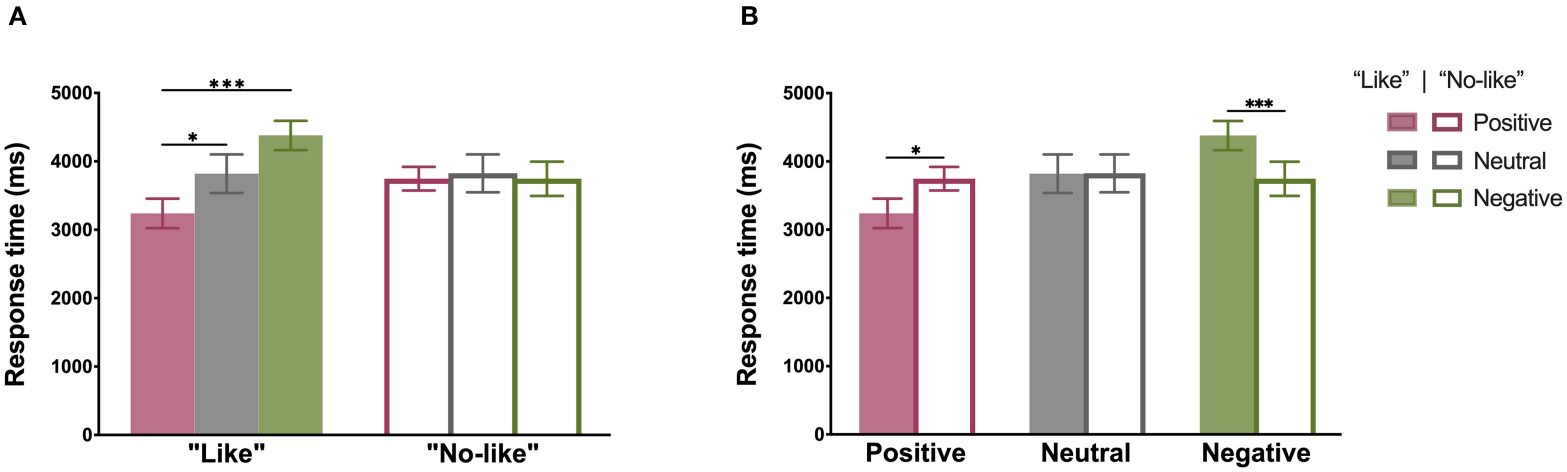
“Like”/“No-like” response times. **(A)** Emotional valence of responses under “Like” vs. “No-like” conditions (positive vs. neutral: Cohen's *d* = −0.31, positive vs. negative: Cohen's *d* = −0.71). **(B)** “Like”/“No-like” effects for different emotional valence conditions (positive: “Like” vs. “No-like”: Cohen's *d* = −0.35; negative: “Like” vs. “No-like”: Cohen's *d* = 0.36). Error bars indicate standard errors. *p < 0.05; ***p < 0.001.

An analysis of the simple effect of whether to “Like” × emotional valence interaction showed that when a participant chose to give “Like” feedback, the response time for positive pictures was shorter than that for the neutral pictures (*p* = 0.036, Cohen's *d* = −0.31) and for the negative pictures (*p* < 0.001, Cohen's *d* = −0.71). Conversely, when the participant chose not to give “Like” feedback, there was no difference in the response time to the positive, neutral, or negative pictures (*ps* > 0.05) (Figure 3A). We further observed that the time taken to “Like” the positive pictures was shorter than the time taken to “No-like” the positive pictures (*p* = 0.035, Cohen's *d* = −0.35). Conversely, the time taken to “Like” the negative pictures was longer than the time taken to “No-like” the negative pictures (*p* < 0.001, Cohen's *d* = 0.36). Meanwhile, there was no difference between the time taken to give, or not give, “Like” feedback to the neutral pictures (*p* = 0.988) (Figure 3B).

## Discussion

“Like” responses of the extrovert and introvert groups to simulated Moments images which had different emotional valences and personal relevance were compared. The Moments images that exhibited positive emotion received more “Likes” and that exhibited negative emotion received less “Likes” compared to the neutral emotion Moments images. In addition, the personally relevant Moments images received more “Likes” than the personally irrelevant Moments images. Meanwhile, the “Like” rates of the extrovert and introvert groups did not differ. However, in the extrovert group, the “Like” rate for the negative-personally irrelevant Moments images was found to positively correlate with neuroticism scores, yet negatively correlated with the conscientiousness scores. Finally, it took participants less time to “Like” the positive Moments images than to decide to not give a “Like.” Conversely, the participants took less time to not click a “Like” for the negative Moments images than to click “Like.” In contrast, there was no difference in the time taken to “Like” or “No-like” the neutral Moments images.

Hedonic gratification motivates individuals to experience positive affects (Meehl, 1975). Correspondingly, greater “Like” feedback was provided for the positive Moments images that were simulated in the present study. While positive emotional stimulation is generally related to hedonic gratification, there are differences. For example, hedonic gratification may be motivated in some individuals by neutral emotional stimulation of something they are interested in (such as a porcelain), or even by negative emotional stimulation (such as a horror film) (Martin, 2019). Functional magnetic resonance imaging (fMRI) has been used to scan the brains of participants when they “Like” photos posted by others on social media. It has been observed that their orbitofrontal cortex (OFC), insula, ventral tegmental area (VTA), striatum (including the pallidum), amygdala, hippocampus, and medial prefrontal cortex (mPFC) in the brain are activated (Sherman et al., 2018). These regions have also been shown to be implicated in the hedonic network (Alexander et al., 2021). Thus, whether “Liking” positive emotional stimulation or negative emotional stimulation, positive hedonic gratification may be experienced. Nevertheless, it is impossible to choose the content to post on Moments according to the personal characteristics of one viewer, either for a specific experimental condition or for an actual poster on Moments. That is why positive emotional images were simulated for this study and were used to detect hedonic gratification of “Liking” behavior in our cohort.

The emotional state of individuals browsing Moments images may be complex and dynamic. If real-time fluctuations in the emotions of a viewer could be monitored, it is anticipated that powerful evidence of hedonic gratification for “Liking” would be obtained. Recently, dynamic Affective Representation Mapping was demonstrated as a technique for measuring real-time fluctuations in emotions as the decision-process unfolds (Heffner et al., 2021). With this tool, the subjective experience of emotion can be precisely and mathematically mapped alongside “Liking” decisions made on social media in future study.

Social gratification of “Liking” behavior is complicated (Kim, 2014; Hayes et al., 2016; Lee et al., 2016; Gan and Chunmei, 2017; Ozanne et al., 2017; Hossain et al., 2019). In the present study, only the desire for interpersonal contact was considered. A multidimensional construct of affiliation motivation proposes that the desire for interpersonal contact is greater among individuals who especially value learning about others and exploring what others are like (Hill, 1987). As a result, the frequency of users sharing personal content has been found to have a direct effect on the frequency of “Likes” they receive from other users on social media (Bartsch et al., 2006; Marengo et al., 2021). In the present study, the participants provided “Like” feedback (as a type of interpersonal contact) for the simulated Moments images that were related to the posters. This result is consistent with a desire for interpersonal contact (namely, social gratification) to serve as a motivation of viewers' “Liking” behavior. Another study also observed that first-person narration alongside images (equivalent to personally relevant Moments images in the present study) gained more “Likes” because they boost a user's motivation to achieve social belonging (Chang et al., 2019). There are other social gratification factors to consider as well. For example, conformity motivation and subjective norms can affect viewers' attitudes to “Like,” “Like” intention, and actual “Liking” behavior (Chin et al., 2015; Dhir et al., 2019; Hossain et al., 2019; Shao and Kwon, 2019). However, these factors are not reflected in the contents of the simulated posts used in the present study. Therefore, it remains for them to be addressed in future experiments.

Extraversion is related to both social gratification and hedonic gratification, since extraversion describes a person's tendency to be sociable and his/her ability to experience positive emotions (Butt and Phillips, 2008). Thus, it is not surprising that extraversion is related to “Liking” behavior on social media (Kabadayi and Price, 2014; Lee et al., 2016). However, similar to a previous study, we did not find a relationship between extraversion and “Liking” behavior (Li and Wang, 2021). A possible reason for this result is that both introverts and extroverts benefit from “Liking” behavior for different reasons. For example, introverts may build up their social relations with “Liking” behavior, while extroverts may provide “Like” feedback for social enhancement (Zywica and Danowski, 2008).

To simulate the actual response of users when they browse Moments, the participants in our study were not asked to respond as quickly as possible. Consequently, the time taken to make a “Like”/“No-like” choice was analyzed. It was observed that “Like” feedback was provided at a faster rate than “No-like” feedback for positive emotional content, and was slower for negative emotional content. Critical social cognitive processing that is responsible for this dissociation between “Liking” and “No-liking” behavior may involve the theory of mind (ToM). ToM is responsible for the comprehension and representation of other's beliefs, intentions, emotions, and feelings, and for predicting the behavior of others (Premack and Woodruff, 1978; Frith and Frith, 2006; Wu et al., 2020). When providing feedback to others, a user may think about how this specific poster may react upon receiving this feedback (Meshi et al., 2015). A poster, the mutual friends of a poster, and a viewer can check “Like” feedback that is provided. Thus, the viewer who clicks “Like” or “No-like” for a particular post may potentially infer the state of mind of the poster. For “Liking” positive contents, ToM may encompass an attractive force. However, ToM may encompass a repulsive force for “Like” feedback for negative content. It is possible that the task of “Liking” on Moments may represent an effective paradigm for detecting cognitive vs. affective ToM.

As mentioned above, the results of the present study provide further insight into possible reasons why viewers “Like” content on social media, with hedonic and social gratification identified as factors. The present results also suggest an effective paradigm to investigate cognitive vs. affective ToM. Regarding practical implications of the present study, a content strategy to attract more “Likes” on social media is demonstrated for both individuals and commercial users. A recent study demonstrated that the frequency of “Likes” received by others positively related with perceived happiness (Marengo et al., 2021). Conversely, feelings of exclusion have been reported when “Likes” are not received from close relations or socially superior network members (Hayes et al., 2018). For brand marketers, consumers' “Likes” can not only enhance brand connections and customer engagements, but can also positively affect online brand endorsement (Hoffman and Fodor, 2010; Malhotra et al., 2013; Bernritter et al., 2016). Overall, it is clear that individuals and commercial users should effectively present on social media to increase “Likes” received.

The current study has certain limitations which should be addressed in future research. First, the effect of hedonic and social gratification on “Liking” behavior on actual social media could be further investigated. It has been reported that the affective state in the Ultimatum Game is higher for unfair offers, and is associated with the rejection of unfair offers. More importantly, this pattern has only been observed for offers proposed by human conspecifics, not for offers generated by computers (van't Wout et al., 2006). In the present study, we attempted to simulate the details of Moments content as realistically as possible. However, our participants are presented with a computer that they know no one is behind. Consequently, their “Liking” behavior may not be the same as that on actual Moments. A second consideration is that additional studies should be conducted to test other factors that may influence “Liking” behavior from the perspective of hedonic and social gratification. For example, the effect of the number of “Likes” that a post has received and the influence of the relationship between the viewer and poster on “liking” behavior could be examined. Finally, a self-report measure of motivation for “Liking” behavior was not included in the present study because we felt that self-reports are uniquely ill-suited to the current research questions. However, it must be acknowledged that there are individual differences in hedonic and social gratification which motivate “Liking” behavior.

## Conclusion

The results of the current study demonstrate that “Liking” behavior may be motivated by hedonic and social gratification. The present results also demonstrate effective content strategies for individual and commercial users to help attract a greater number of “Likes” on social media. In particular, content should have a positive emotional aspect and should be personally relevant.

## Data Availability Statement

The original contributions presented in the study are included in the article/Supplementary Material, further inquiries can be directed to the corresponding author.

## Ethics Statement

The studies involving human participants were reviewed and approved by Academic Committee of School of Education and Psychology, Southwest University for Nationalities. The patients/participants provided their written informed consent to participate in this study.

## Author Contributions

CZ: conceptualization, methodology, supervision, writing-original draft, writing-review & editing, re-constructing the theoretical framwork, re-writing introduction and discussion. XS: investigation, data curation, and writing-original draft. JL: formal analysis and writing-review & editing. TD, YC, and SY: resources and investigation. All authors contributed to the article and approved the submitted version.

## Funding

This work was supported by a Fundamental Research Fund for the Central Universities [Grant Number 2015NZYQN83] and a National College Students Innovation and Entrepreneurship Training Program [Grant Number X202010656262].

## Conflict of Interest

The authors declare that the research was conducted in the absence of any commercial or financial relationships that could be construed as a potential conflict of interest.

## Publisher's Note

All claims expressed in this article are solely those of the authors and do not necessarily represent those of their affiliated organizations, or those of the publisher, the editors and the reviewers. Any product that may be evaluated in this article, or claim that may be made by its manufacturer, is not guaranteed or endorsed by the publisher.

## References

[B1] Ajzen I (1991). The theory of planned behavior. Organiz. Behav. Hum. Decis. Processes 50, 179–211. 10.1016/0749-5978(91)90020-T

[B2] Alexander R. Aragon O. R. Bookwala J. Cherbuin N. Gatt J. M. Kahrilas I. J.. (2021). The neuroscience of positive emotions and affect: Implications for cultivating happiness and wellbeing. Neurosci. Biobehav. Rev. 121, 220–249. 10.1016/j.neubiorev.2020.12.002 33307046

[B3] Bai L. Ma H. Huang Y. Luo Y. (2005). The development of native Chinese affective picture system—a pretest in 46 college students. Chinese Ment. Health J. 19, 4–7. 10.3321/j.issn:1000-6729.2005.11.001

[B4] Bartsch A. Mangold R. Viehoff R. Vorderer P. (2006). Emotional gratifications during media use–an integrative approach. Communications 31, 261–278. 10.1515/COMMUN.2006.018

[B5] Bernritter S. F. Verlegh P. W. J. Smit E. G. (2016). Why nonprofits are easier to endorse on social media: the roles of warmth and brand symbolism. J. Interact. Market. 33, 27–42. 10.1016/j.intmar.2015.10.002

[B6] Butt S. Phillips J. G. (2008). Personality and self reported mobile phone use. Comput. Hum. Behav. 24, 346–360. 10.1016/j.chb.2007.01.019

[B7] Chang Y. Li Y. Yan J. Kumar V. (2019). Getting more likes: the impact of narrative person and brand image on customer-brand interactions. J. Acad. Market. Sci. 47, 1027–1045. 10.1007/s11747-019-00632-2

[B8] Chin C. Y. Lu H. P. Wu C. M. (2015). Facebook users' motivation for clicking the “like” button. Soc. Behav. Personal. 43, 579–592. 10.2224/sbp.2015.43.4.579

[B9] Costa P. T. J. McCrae R. R. (1992). Revised NEO Personality Inventory (NEO-PI-R) and NEO Five-Factor Inventory (NEO-FFI) professional manual. Odessa: FL: Psychological Assessment Resources.

[B10] Dhir A. Khalil A. Kaur P. Rajala R. (2019). Rationale for ”liking“ on social networking sites. Soc. Sci. Comput. Rev. 37, 529–550. 10.1177/089443931877914522973420

[B11] Frith C. D. Frith U. (2006). The neural basis of mentalizing. Neuron 50, 531–534. 10.1016/j.neuron.2006.05.001 16701204

[B12] Gan T. Chunmei D. (2017). Understanding WeChat users' liking behavior: an empirical study in China. Comput. Hum. Behav. 68, 30–39. 10.1016/j.chb.2016.11.002

[B13] Gosling S. D. Augustine A. A. Vazire S. Holtzman N. Gaddis S. (2011). Manifestations of personality in online social networks: Self-reported Facebook-related behaviors and observable profile information. Cyberpsychol Behav. Soc. Network. 14, 483–488. 10.1089/cyber.2010.0087 21254929 PMC3180765

[B14] Hayes R. A. Carr C. T. Wohn D. Y. (2016). One click, many meanings: Interpreting paralinguistic digital affordances in social media. J. Broadcast. Electron. Media 60, 171–187. 10.1080/08838151.2015.1127248

[B15] Hayes R. A. Wesselmann E. D. Carr C. T. (2018). When nobody ”likes“ you: Perceived ostracism through paralinguistic digital affordances within social media. Soc. Media + Society 4, 1–12. 10.1177/2056305118800309

[B16] Heffner J. Son J.-Y. FeldmanHall O. (2021). Emotion prediction errors guide socially adaptive behaviour. Nat. Hum. Behav. 5, 1391–U1157. 10.1038/s41562-021-01213-6 34667302 PMC8544818

[B17] Hill C. A (1987). Affiliation motivation: people who need people . but in different ways. J. Personal. Soc. Psychol. 52, 1008–1018. 10.1037/0022-3514.52.5.1008 3585697

[B18] Hoffman D. L. Fodor M. (2010). Can you measure the ROI of your social media marketing? Mit Sloan Manage. Rev. 52, 41–49

[B19] Hossain M. A. Kim M. Jahan N. (2019). Can ”liking“ behavior lead to usage intention on Facebook? uses and gratification theory perspective. Sustainability 11, 1–13. 10.3390/su11041166

[B20] Kabadayi S. Price K. (2014). Consumer – brand engagement on Facebook: liking and commenting behaviors. J. Res. Interact. Market. 8, 203–223. 10.1108/JRIM-12-2013-0081

[B21] Kim J. W (2014). Scan and click: The uses and gratifications of social recommendation systems. Comput. Hum. Behav. 33, 184–191. 10.1016/j.chb.2014.01.028

[B22] Lee S. Y. Hansen S. S. Lee J. K. (2016). What makes us click ”like“ on Facebook? examining psychological, technological, and motivational factors on virtual endorsement. Comput. Commun. 73, 332–341. 10.1016/j.comcom.2015.08.002

[B23] Li H. Wang X. T. (2021). Cyber-personality and liking expression: a study from WeChat users in China. Front. Psychol. 12,6040. 10.3389/fpsyg.2021.626040 34305702 PMC8295648

[B24] Luo J. Dai X. (2011). Meta-analysis of big-five factor personality tests in China. Chinese J. Clinic. Psychol. 19, 740–752. 10.16128/j.cnki.1005-3611.2011.06.008

[B25] Malhotra A. Malhotra C. K. See A. (2013). How to create brand engagement on Facebook. Mit Sloan Manage. Rev. 54, 18–20 10.5005/jp/books/12036_3

[B26] Marengo D. Montag C. Sindermann C. Elhai J. D. Settanni M. (2021). Examining the links between active Facebook use, received likes, self-esteem and happiness: A study using objective social media data. Telematics Informat. 58,101523. 10.1016/j.tele.2020.101523

[B27] Martin G. N (2019). (Why) do you like scary movies? a review of the empirical research on psychological responses to horror films. Front. Psychol. 10,23. 10.3389/fpsyg.2019.02298 31681095 PMC6813198

[B28] Mccrae R. R. Costa P. T. (2004). A contemplated revision of the NEO five-factor inventory. Personal. Individ. Differen. 36, 587–596. 10.1016/S0191-8869(03)00118-1

[B29] Meehl P. E (1975). Hedonic capacity: some conjectures. Bull. Menninger Clinic 39, 295–307 1156704

[B30] Meshi D. Tamir D. I. Heekeren H. R. (2015). The emerging neuroscience of social media. Trends Cogn. Sci. 19, 771–782. 10.1016/j.tics.2015.09.004 26578288

[B31] Ozanne M. Navas A. C. Mattila A. S. Hoof H. B. V. (2017). An Investigation Into Facebook “Liking” behavior an exploratory study. Social Media + Society 3, 1–12. 10.1177/2056305117706785

[B32] Papacharissi Z. Mendelson A. (2011). Toward a new(er) sociability: Uses, gratifications and social capital on Facebook. London: Taylor and Francis, 212–230.

[B33] Premack D. Woodruff G. (1978). Does the chimpanzee have a theory of mind. Behav. Brain Sci. 1, 515–526. 10.1017/S0140525X0007651230886898

[B34] Ruggiero T. E (2000). Uses and gratifications theory in the 21st century. Mass Commun. Soc. 3, 3–37. 10.1207/S15327825MCS0301_02

[B35] Shao C. Kwon K. H. (2019). Clicks intended: an integrated model for nuanced social feedback system uses on Facebook. Telematics Informatics 39, 11–24. 10.1016/j.tele.2018.12.003

[B36] Sherman L. E. Hernandez L. M. Greenfield P. M. Dapretto M. (2018). What the brain 'Likes': neural correlates of providing feedback on social media. Soc. Cogn. Affect. Neurosci. 13, 699–707. 10.1093/scan/nsy051 29982823 PMC6121147

[B37] Sumner E. M. Ruge-Jones L. Alcorn D. (2018). A functional approach to the Facebook Like button: An exploration of meaning, interpersonal functionality, and potential alternative response buttons. New Media Soc. 20, 1451–1469. 10.1177/1461444817697917

[B38] Sun Y. Liu D. Shen X. L. Zhang X. Wang N. (2016). “Understanding the factors affecting users' like intentions in social network services: a multi-dimensional value perspective*,”* in Paper presented at the 49th Hawaii International Conference on System Sciences.

[B39] Tazghini S. Siedlecki K. L. (2013). A mixed method approach to examining Facebook use and its relationship to self-esteem. Comput. Hum. Behav. 29, 827–832. 10.1016/j.chb.2012.11.010

[B40] van't Wout M. Kahn R. S. Sanfey A. G. Aleman A. (2006). Affective state and decision-making in the Ultimatum Game. Experiment. Brain Res. 169, 564–568. 10.1007/s00221-006-0346-5 16489438

[B41] Wang N. Sun Y. Shen X.-L. Liu D. Zhang X. (2019). Just being there matters Investigating the role of sense of presence in Like behaviors from the perspective of symbolic interactionism. Internet Res. 29, 60–81. 10.1108/IntR-08-2017-0299

[B42] Wilson K. Fornasier S. White K. M. (2010). Psychological predictors of young adults' use of social networking sites. Cyberpsychol. Behav. Soc. Network. 13, 173–177. 10.1089/cyber.2009.0094 20528274

[B43] Wu H. Y. Liu X. Hagan C. C. Mobbs D. (2020). Mentalizing during social interAction: a four component model. Cortex 126, 242–252. 10.1016/j.cortex.2019.12.031 32092493 PMC7739946

[B44] Yao R. Liang L. (2010). Analysis of the application of simplified NEO-FFI to undergraduates. Chinese J. Clinic. Psychol. 18, 457–459. 10.16128/j.cnki.1005-3611.2010.04.024

[B45] Zhao H. Zhang M. (2020). The role of guanxi and positive emotions in predicting users' likelihood to click the like button on WeChat. Front. Psychol. 11,1736. 10.3389/fpsyg.2020.01736 32793067 PMC7387579

[B46] Zywica J. Danowski J. (2008). The faces of Facebookers: Investigating social enhancement and social compensation hypotheses; predicting Facebook and offline popularity from sociability and self- esteem, and mapping the meanings of popularity with semantic networks. J. Comput. Mediated Commun. 14, 1–34. 10.1111/j.1083-6101.2008.01429.x

